# Targeting Death Receptor TRAIL-R2 by Chalcones for TRAIL-Induced Apoptosis in Cancer Cells

**DOI:** 10.3390/ijms131115343

**Published:** 2012-11-20

**Authors:** Ewelina Szliszka, Dagmara Jaworska, Małgorzata Kłósek, Zenon P. Czuba, Wojciech Król

**Affiliations:** Chair and Department of Microbiology and Immunology, Medical University of Silesia in Katowice, Jordana 19, 41-808 Zabrze, Poland; E-Mails: eszliszka@sum.edu.pl (E.S.); djaworska@sum.edu.pl (D.J.); klosekmalgorzata@gmail.com (M.K.); zczuba@sum.edu.pl (Z.P.C.)

**Keywords:** TRAIL, chalcones, apoptosis, death receptors, cancer cells, chemoprevention

## Abstract

Tumor necrosis factor-related apoptosis-inducing ligand (TRAIL) induces apoptosis in cancer cells without toxicity to normal cells. TRAIL binds to death receptors, TRAIL-R1 (DR4) and TRAIL-R2 (DR5) expressed on cancer cell surface and activates apoptotic pathways. Endogenous TRAIL plays an important role in immune surveillance and defense against cancer cells. However, as more tumor cells are reported to be resistant to TRAIL mediated death, it is important to search for and develop new strategies to overcome this resistance. Chalcones can sensitize cancer cells to TRAIL-induced apoptosis. We examined the cytotoxic and apoptotic effects of TRAIL in combination with four chalcones: chalcone, isobavachalcone, licochalcone A and xanthohumol on HeLa cancer cells. The cytotoxicity was measured by MTT and LDH assays. The apoptosis was detected using annexin V-FITC staining by flow cytometry and fluorescence microscopy. Death receptor expression was analyzed using flow cytometry. The decreased expression of death receptors in cancer cells may be the cause of TRAIL-resistance. Chalcones enhance TRAIL-induced apoptosis in HeLa cells through increased expression of TRAIL-R2. Our study has indicated that chalcones augment the antitumor activity of TRAIL and confirm their cancer chemopreventive properties.

## 1. Introduction

The death ligand TRAIL (tumor necrosis factor-related apoptosis-inducing ligand), a member of the TNF superfamily is recognized as a promising anticancer agent due to selective killing of cancer cells without toxicity to normal cells [1]. TRAIL plays an important role in immune surveillance and defense mechanisms against tumor cells. The death ligand is expressed on the T lymphocytes, natural killer cells, dendritic cells, neutrophils, monocytes or macrophages [2,3]. Membrane-bound TRAIL can be cleaved from the cell surface into a soluble secreted form [1,4]. Endogenous TRAIL triggers apoptosis in cancer cells via receptor-mediated death through interaction with the death receptors (DRs) [5]. There are two agonistic transmembrane receptors, TRAIL-R1/DR4 and TRAIL-R2/DR5, which bind ligands by extracellular domains. The death receptors contain complete and functional cytoplasmic death domains responsible for the activation of apoptotic pathway in cancer cells [5,6]. The downstream signaling cascade includes receptor oligomerization, recruitment of the adaptor molecule FADD (Fas-associated death domain) with formation of the DISC (death inducing signaling complex), activation of initiator caspases, cleavage of effector caspases and finally DNA fragmentation [6–8].

However, some tumor cells are resistant to TRAIL-mediated death [8–10]. Failure to undergo apoptosis has been implicated in the resistance of cancer cells to TRAIL surveillance and therefore in tumor development [11]. The expression of the death receptors on cancer cell surface is involved in TRAIL-resistance [5,6,9]. TRAIL-R2 called “KILLER” receptor is a crucial player in the transduction of apoptotic signaling in cancer cells derived from solid tumors [4,12]. We and others have shown that TRAIL-resistant cancer cells can be sensitized by polyphenols [13–16].

Chalcones (1,3-diphenyl-2-propen-1-ones) represent an important group of the polyphenolic family widespread in various spices, fruits, vegetables, tea or beer [17–19]. Isobavachalcone is found in various medicinal plants, such as *Psoralea corylifolia*, *Angelica keiskei* and *Broussonetia papyrifera*. Licochalcone A is isolated from root of herb named licorice (*Glycyrrhiza glabra*, *Glycyrrhiza uralensis*). Xanthohumol is identified in hops (*Humulus lupulus*), an essential raw material for beer brewing [20–25]. Chemically, they are open-chain flavonoids bearing two aromatic rings joined by a three-carbon α,β-unsaturated carbonyl system [25–29].

Chemoprevention is a method of tumor control in which malignancy is prevented or reversed by nutritional or pharmacological intervention using natural or synthetic substances [16,30]. The role of polyphenols in cancer prevention has been confirmed in numerous laboratory, clinical and epidemiological studies [16,31]. The *in vitro* and *in vivo* experiments provide the evidence that chalcones target the multistep carcinogenetic process by scavenging reactive oxygen species, regulating cell proliferation, inducing apoptosis, inhibiting tumor invasion and metastasis, blocking angiogenesis and affecting metabolism of xenobiotics [17,18,32].

**Figure 1:**
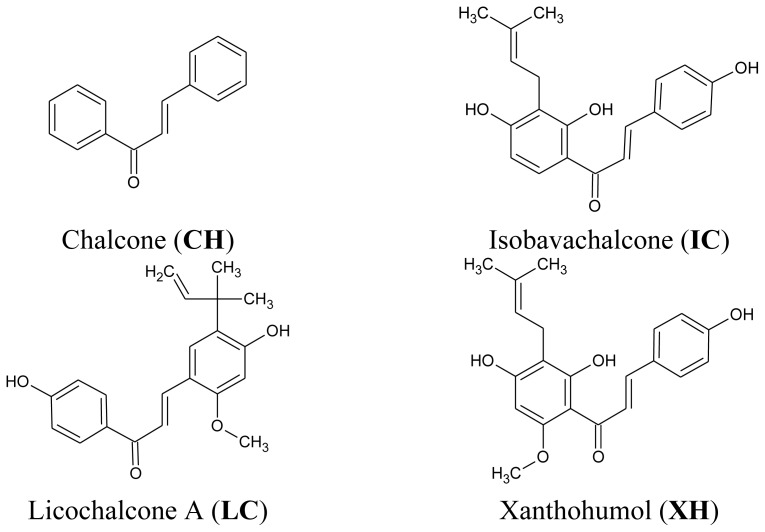
Chemical structures of the studied chalcones.

Our previous findings demonstrated that chalcones and dihydrochalcones augment TRAIL-mediated apoptosis in LNCaP prostate cancer cells [33,34]. The present study is a continuation of these investigations and exploration of the mechanism of action exhibited by chalcones on TRAIL-mediated apoptosis. Now we examine the cytotoxic and apoptotic effects of TRAIL in combination with four chalcones: chalcone, isobavachalcone, licochalcone A and xanthohumol on HeLa cervical cancer cells. The chemical structures of the tested compounds are shown in Figure 1. We report the molecular mechanism by which these chalcones enhance TRAIL-induced apoptosis in cancer cells. The obtained results suggest that the overcoming of TRAIL-resistance by chalcones may be one of the mechanisms responsible for their cancer chemopreventive activities.

## 2. Results and Discussion

### 2.1. Cytotoxic and Apoptotic Activities of TRAIL in HeLa Cancer Cells

TRAIL is an important component of the immune defense and powerful inducer of apoptosis in cancer cells [35]. Active avoidance of apoptosis promoting cancer cells survival is one of the hallmarks of tumor development [1,4,36]. Many type of cancer cell lines are TRAIL-resistant [9,16,37].

We and others have demonstrated that the HeLa cell line is also resistant to TRAIL-mediated death [13,15,38,39]. Recombinant human TRAIL used in our study is a soluble protein based on a natural endogenous ligand [38,39]. TRAIL at the concentration of 100 ng/mL induced 9.42% ± 0.9% cell death. The cytotoxicity was measured by MTT assay. This ligand causes the cytotoxic effect in cancer cells via the apoptotic route [13]. The necrotic cell death percentage of HeLa cells examined by lactate dehydrogenase assay, by flow cytometry with propidium iodide and by fluorescence microscopy with Ethidium Homodimer III was near 0%. The apoptotic activity of TRAIL at the concentration of 100 ng/mL was 14.4% ± 0.9%. TRAIL concentrations of 200 ng/mL or higher did not significantly increase the cytotoxic and apoptotic effects on HeLa cells.

### 2.2. Cytotoxic and Apoptotic Activities of Chalcones in HeLa Cancer Cells

Chalcones have been recently subject of great interest for their pharmacological activities, such as anti-inflammatory, antioxidant, anticancer and chemopreventive properties. Therefore, the application of natural or synthetic chalcones is becoming increasingly recognized as an effective strategy in cancer prevention and therapy [17,18,27–29].

**Figure 2:**
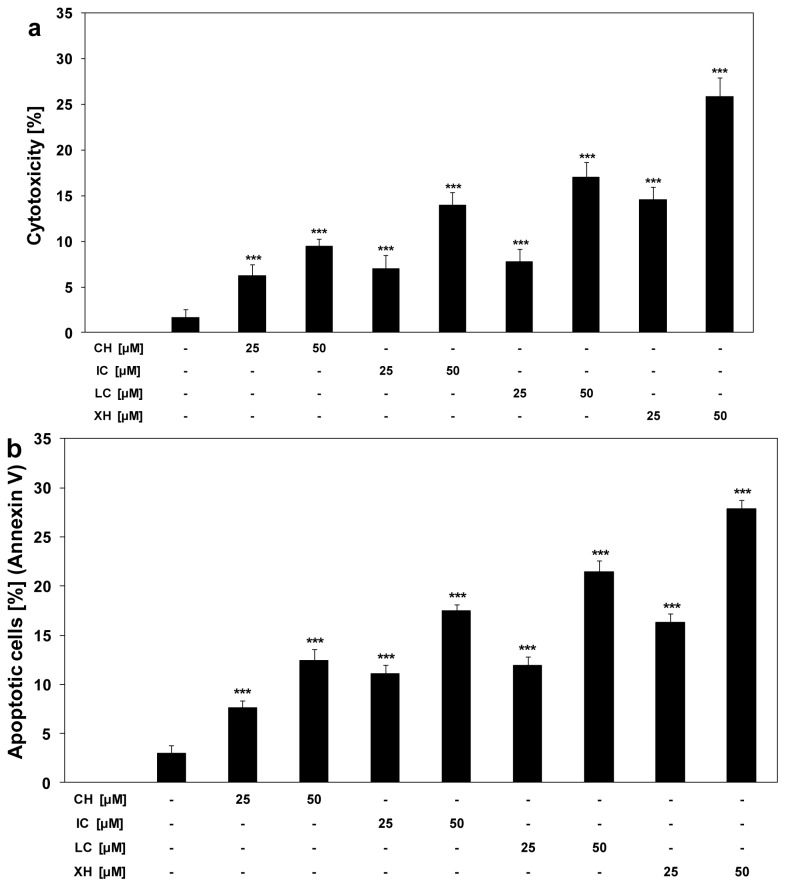
Cytotoxic and apoptotic effects of chalcones in HeLa cancer cells. The cells were incubated for 24 h with chalcones at the concentrations of 25 μM and 50 μM. The values represent mean ± SD of three independent experiments performed in quadruplicate (***p < 0.001 compared with control). (**a**) Cytotoxic activity of chalcones in HeLa cells. The percentage of cell death was measured by MTT cytotoxicity assay; (**b**) Apoptotic activity of chalcones in HeLa cells. Detection of apoptotic cell death by annexin V-FITC staining using flow cytometry.

We tested anticancer activity of chalcones at the concentrations of 25 μM and 50 μM against HeLa cells. The compounds induce cytotoxic and apoptotic effects in a dose-dependent manner. The cytotoxicity of chalcones in HeLa cells was: 6.3% ± 1.2%–9.4% ± 0.9% cell death for chalcone, 7.0% ± 1.4%–13.9% ± 1.4% cell death for isobavachalcone, 7.8% ± 1.4%–17.4% ± 1.7% cell death for licochalcone A, 14.5% ± 1.4%–25.8% ± 2.1% cell death for xanthohumol (Figure 2A).

Our results indicate that this cytotoxic effect was mediated through apoptosis. The percentage of necrotic cells examined by lactate dehydrogenase assay and fluorescence microscopy with Ethidium Homodimer III was near 0%. Chalones cause apoptosis in HeLa cells: 7.6% ± 0.7%–12.5% ± 1.1% cell death for chalcone, 11.1% ± 0.9%–17.5% ± 0.6% cell death for isobavachalcone, 11.9% ± 0.9%–21.4% ± 1.2% cell death for licochalcone A, 16.3% ± 0.8%–27.8% ± 0.9% cell death for xanthohumol (Figure 2B).

Chalcone arrests cell cycle and triggers apoptosis in T24, HT1376 bladder cancer cells and MCF7 and MDA-MD231 breast cancer cells [40,41]. Isobavachalcone shows an anti-proliferative and pro-apoptotic activities against OVAR8 ovarian and PC3 prostate cancer cells, and against IMR32, NB39 neuroblastoma cells [42,43]. Licochalcone A blocks cell cycle progression and induces apoptosis in PC3 prostate cancer cells [23]. Xanthohumol exhibits growth inhibitory and apoptosis-inducing effects on OVAR and SKOV3 ovarian cancer cell lines and on LNCaP, DU145 and PC3 prostate cancer cell lines [44,45].

### 2.3. Cytotoxic and Apoptotic Activities of TRAIL in Combination with Chalcones in HeLa Cancer Cells

Induction of cancer cell-specific apoptosis *via* activation of TRAIL signaling has become an important focus of cancer research [1,4]. Deregulation of the TRAIL-mediated apoptotic pathway is significant in the initiation and progression of malignancy [1,11,46]. However, as more tumor cells are reported to be resistant to TRAIL-induced death, it is important to identify compounds that potently restore TRAIL-sensitivity. Chalcones have been shown to sensitize cancer cells to TRAIL-induced apoptosis [33,34,47–50].

**Figure 3:**
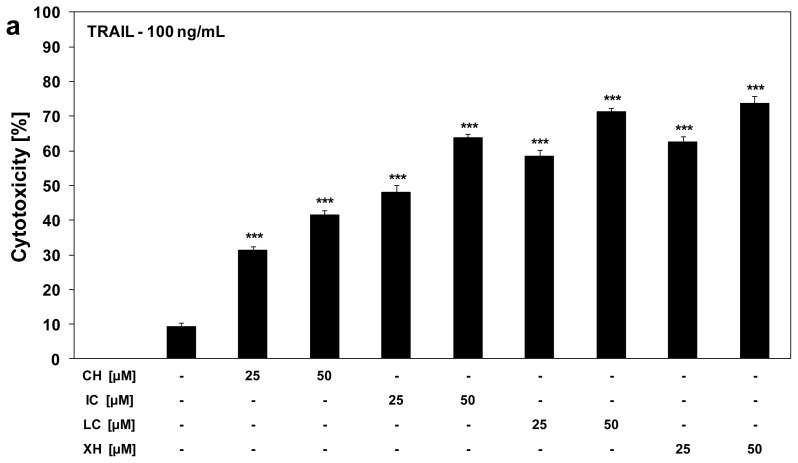

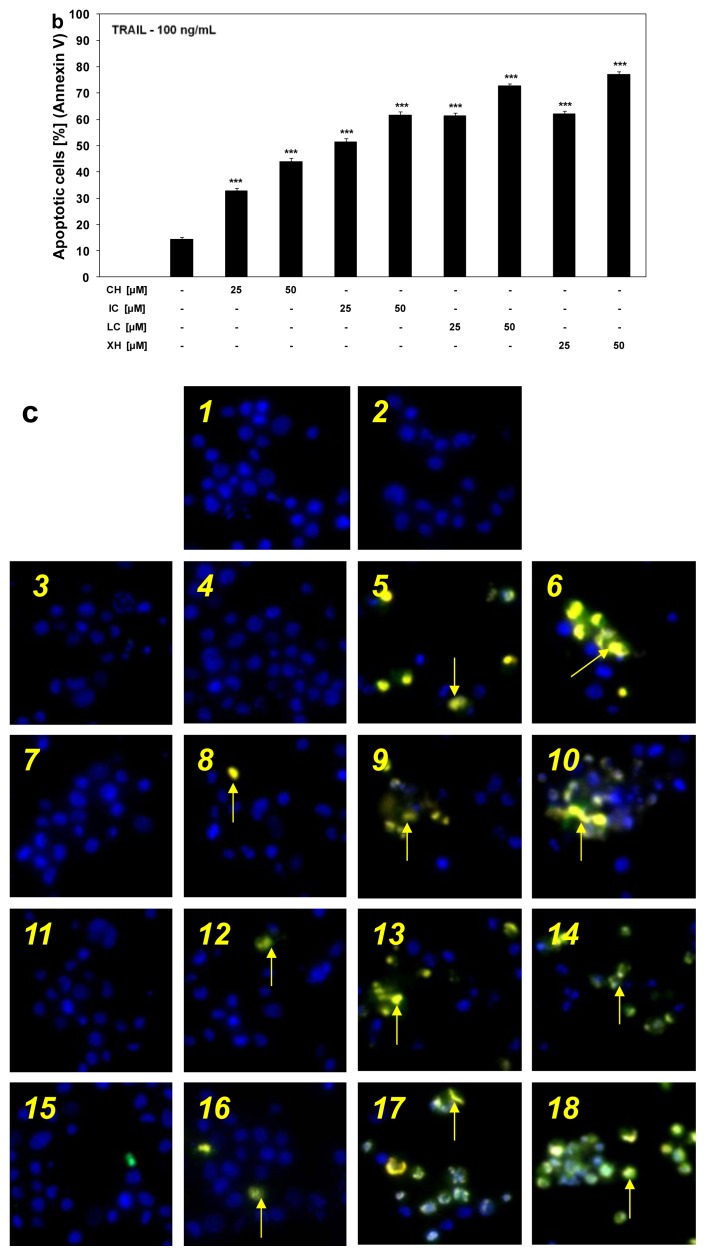
Cytotoxic and apoptotic effects of tumor necrosis factor-related apoptosis-inducing ligand (TRAIL) in combination with chalcones in HeLa cancer cells. The cells were incubated for 24 h with TRAIL at the concentration of 100 ng/mL and chalcones at the concentrations of 25 μM and 50 μM. The values represent mean ± SD of three independent experiments performed in quadruplicate (*** *p* < 0.001 compared with TRAIL). (**a**) Co-treatment of TRAIL with chalcones induced cytotoxicity in HeLa cells. The percentage of cell death was measured by MTT cytotoxicity assay; (**b**) Co-treatment of TRAIL with chalcones induced apoptosis in HeLa cells. Detection of apoptotic cell death by annexin V-FITC and propidium iodide staining using flow cytometry; (**c**) Co-treatment of TRAIL with chalcones induced apoptosis in HeLa cells: (**1**) control cells; (**2**) cells incubated with 100 ng/mL TRAIL; (**3**) cells incubated with 25 μM chalcone; (**4**) cells incubated with 50 μM chalcone; (**5**) cells incubated with 100 ng/mL TRAIL and 25 μM chalcone; (**6**) cells incubated with 100 ng/mL TRAIL and 50 μM chalcone; (**7**) cells incubated with 25 μM isobavachalcone; (**8**) cells incubated with 50 μM isobavachalcone; (**9**) cells incubated with 100 ng/mL TRAIL and 25 μM isobavachalcone; (**10**) cells incubated with 100 ng/mL TRAIL and 50 μM isobavachalcone; (**11**) cells incubated with 25 μM licochalcone A; (**12**) cells incubated with 50 μM licochalcone A; (**13**) cells incubated with 100 ng/mL TRAIL and 25 μM licochalcone A; (**14**) cells incubated with 100 ng/mL TRAIL and 50 μM licochalcone A; (**15**) cells incubated with 25 μM xanthohumol; (**16**) cells incubated with 50 μM xanthohumol; (**17**) cells incubated with 100 ng/mL TRAIL and 25 μM xanthohumol; (**18**) cells incubated with 100 ng/mL TRAIL and 50 μM xanthohumol. Detection of apoptotic cell death by fluorescence microscopy using annexin V-FITC, Ethidium Homodimer III and Hoechst 33342 staining. The healthy cells (stained with Hoechst 33342) emitted blue fluorescence and apoptotic cells (stained with Annexin V-FITC and Hoechst 33342) emitted green and blue fluorescence (indicated by arrows). Cells undergoing apoptosis showed nuclei shrinkage, chromatin condensation and nuclei fragmentation.

We investigated the cytotoxic and apoptotic effects of TRAIL in combination with chalcones on HeLa cancer cells. The cytotoxicity of TRAIL at the concentration of 100 ng/mL in combination with chalcones at the concentrations of 25 μM and 50 μM in HeLa cells was significantly increased to 31.3% ± 1.3%–41.6% ± 1.2% cell death for chalcone, to 48.2% ± 1.9%–63.8% ± 1.1% cell death for isobavachalcone, to 58.6% ± 1.6%–71.3% ± 1.2% cell death for licochalcone A, and to 62.7% ± 1.6%–73.8% ± 2.0% cell death for xanthohumol in comparison to TRAIL alone (Figure 3A). Chalcones cooperate with TRAIL to induce apoptosis in cancer cells (Figure 3B).

When HeLa cells were treated with the same concentrations of TRAIL and tested compounds, the percentage of apoptotic cells determined by annexin V-FITC staining using flow cytometry was elevated to 32.9% ± 1.1%–44.1% ± 1.3% for chalcone, to 51.5% ± 1.2%–61.7% ± 1.2% for isobavachalcone, to 61.4% ± 1.1%–72.7% ± 1.0% for licochalcone A, and to 62.0% ± 1.2%–77.1% ± 1.2% for xanthohumol.

Chalcones overcome the TRAIL-resistance of HeLa cells and markedly increase anticancer effect of death ligand. The annexin V-FITC staining visualized by fluorescence microscopy, supports the hypothesis that the apoptotic activity of TRAIL was augmented by chalcones in cancer cells (Figure 3C). The necrotic cell death percentage of HeLa cells examined by lactate dehydrogenase assay, Apoptest-FITC and Apoptotic & Necrotic & Healthy Cells Quantification Kit was near zero. Isobavachalcone, licochalcone A and xanthohumol exhibited strong cytotoxic and apoptotic effects in combination with TRAIL against cancer cells. Sensitization of cancer cells to TRAIL-mediated death by chalcones suggests the potential role of these compounds in augmentation of anticancer immune defense in which endogenous TRAIL takes part.

In our previous tests we showed that chalcone, licochalcone A, isobavachalcone, xanthohumol reversed TRAIL-resistance in LNCaP prostate cancer cells (however, we did not study the mechanism in depth). Three similar studies with flavokawain B, isoliquiritigenin and butein demonstrated that chalcones synergistically with TRAIL mediate apoptosis in malignant tumor cells principally by induction of death receptor TRAIL-R2 (DR5) [47–50]. Tang *et al.* show the sensitization of PC3 prostate cancer to TRAIL-induced apoptosis by flavokawain B via increase of TRAIL-R2 and pro-apoptotic protein Bim expression and decrease of anti-apoptotic proteins XIAP and survivin in DU145 and PC3 prostate cancer cell [50]. Yoshida *et al.* indicated that isoliquiritigenin overcomes TRAIL-resistance in HT29 human colon cancer cells through up-regulation of TRAIL-R2 expression [47]. Kim describes the augmentation of TRAIL-mediated apoptosis in U937 human leukemia incubated with butein by increase of TRAIL-R2 expression and the caspase-3 activation [48]. Moon *et al.* confirm the role of up-regulation of TRAIL-R2 expression caused by butein in enhancement of TRAIL-induced apoptosis in Hep3B hapatoma cells [49]. Strategies for overcoming resistance to TRAIL-mediated apoptosis include direct targeting of death receptor expression [6,12].

### 2.4. Chalcones Enhance TRAIL-Induced Apoptosis in HeLa Cancer Cells through Up-Regulation of TRAIL-R2

**Figure 4:**
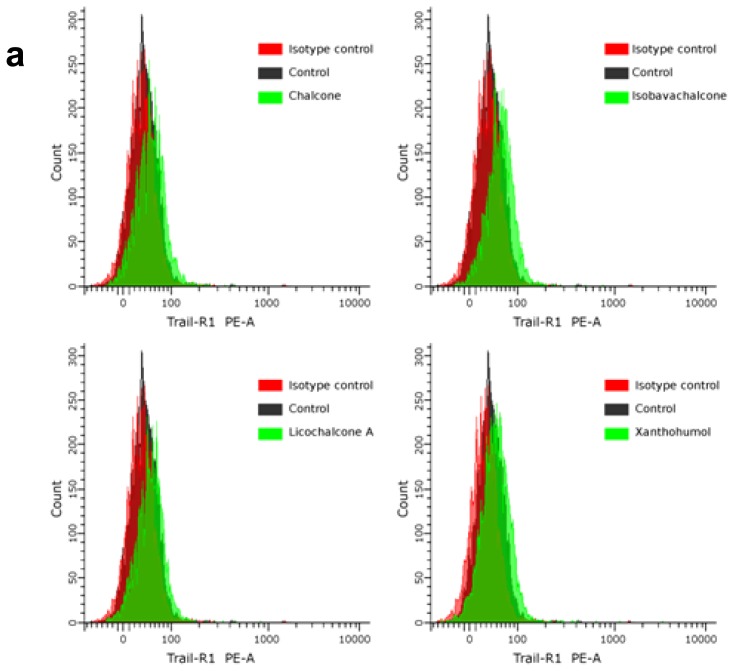

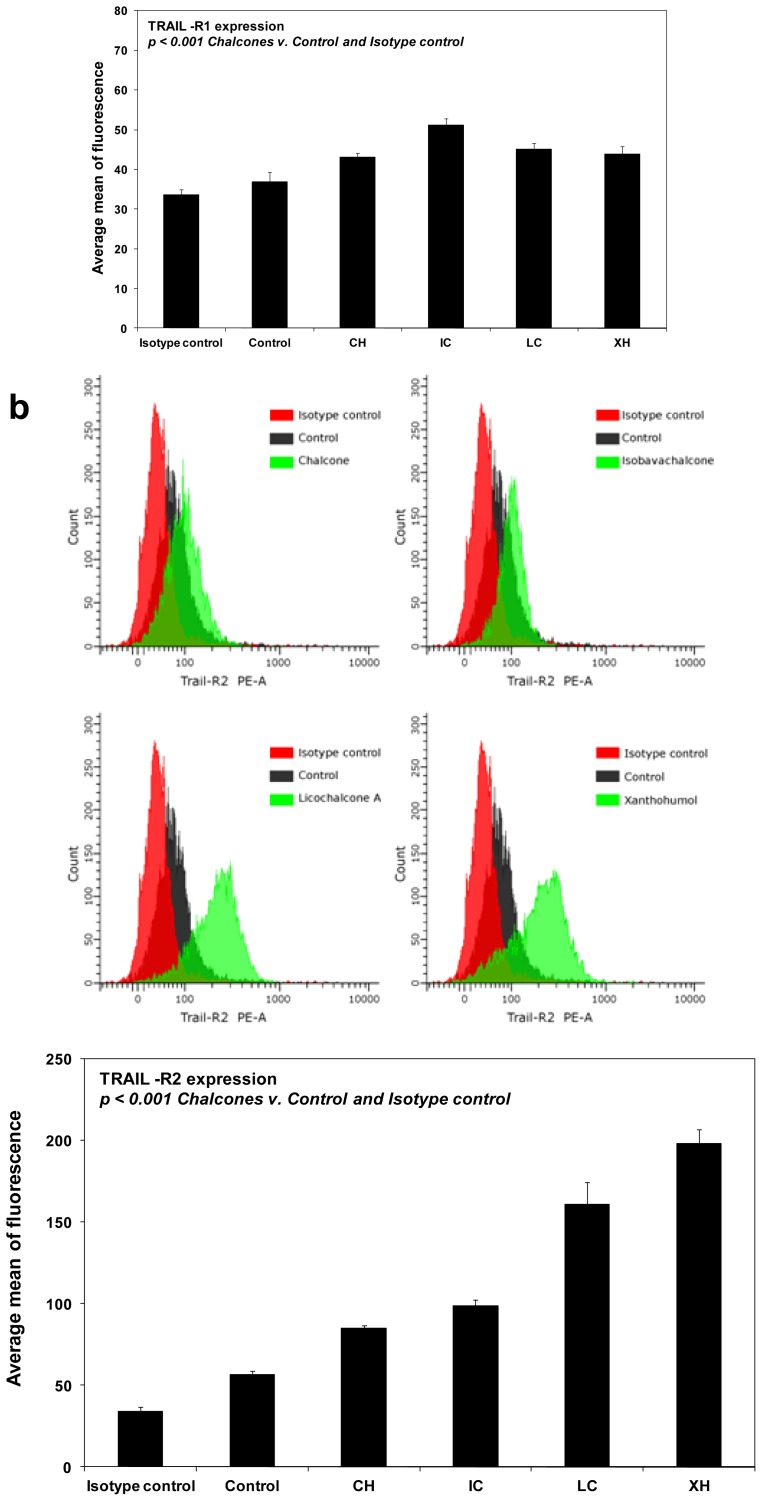
Effects of chalcones on death receptor expression in HeLa cancer cells. The cells were incubated for 24 h with compounds at the concentration of 25 μM. The surface expression of (**a**) TRAIL-R1 and (**b**) TRAIL-R2 on cancer cell was determined by flow cytometry. The values represent mean ± SD of three independent experiments performed in quadruplicate.

Molecular insights into the regulation of apoptosis and defects in apoptosis signaling will help define the TRAIL-resistance of cancer cells and will provide new approaches for preventive or therapeutic intervention against tumors. TRAIL is one of several members of the TNF superfamily that induce apoptosis through engagement of death receptors [12]. Expression levels of TRAIL-R1/DR4 and/or TRAIL-R2/DR5 on the cancer cell surface may play a critical role in intensity and/or duration of death receptor-mediated signaling in response to death ligands [5,6]. We analyzed the expression of TRAIL-R1 and TRAIL-R2 proteins in HeLa cells after 24-hour treatment with chalcones at the concentration of 25 μM by flow cytometry (Figure 4).

Chalcones significantly increase TRAIL-R2 protein levels on the cell surface. The compounds have a little, but also significant effect on TRAIL-R1 protein levels. To determine that the induction of apoptosis by the combination of TRAIL and chalcones was mediated through death receptor TRAIL-R2, we used the TRAIL-R2/Fc chimera protein, which has a dominant negative function against TRAIL-R2.

**Figure 5:**
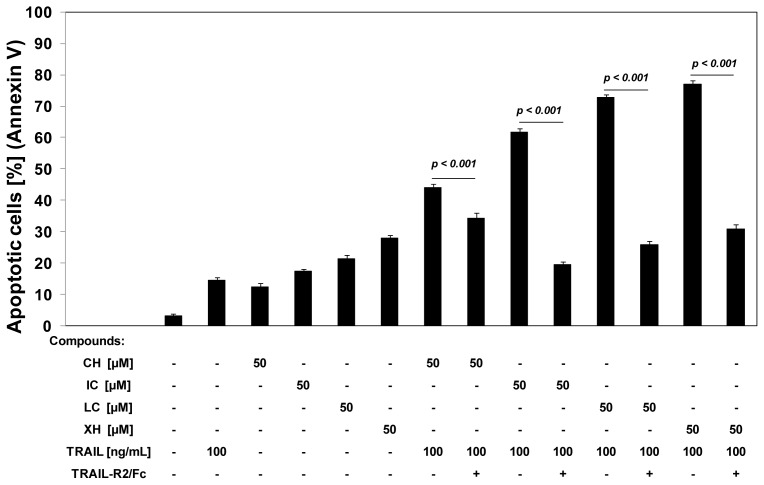
TRAIL-R2/Fc chimera block apoptosis induced by the combination of TRAIL and chalcones in HeLa cancer cells. The cells were incubated for 24 h with 100 ng/mL TRAIL and/or 50 μM chalcones with or without 1 μg/mL TRAIL-R2/Fc chimera proteins. Apoptotic cell death was detected by annexin V-FITC staining using flow cytometry. The values represent mean ± SD of three independent experiments performed in duplicate.

The TRAIL-R2/Fc efficiently blocked apoptosis caused by the co-treatment of TRAIL with chalcones (Figure 5). These data suggested that chalcones sensitize cancer cells to TRAIL through the extrinsic (receptor) apoptotic pathway via affecting TRAIL-R2.

TRAIL-R2 up-regulation has become the most important strategy employed by polyphenols to sensitize TRAIL-resistant cancer cells to TRAIL-mediated apoptosis. Many recent studies have revealed that TRAIL-R2 may play a more prominent role than TRAIL-R1 in TRAIL-mediated apoptotic signaling, especially in cancer cells derived from solid tumors [6,12,51]. Numerous findings support the fact that TRAIL-resistance in cancer cells can be reversed by modulation of TRAIL-R2 by polyphenols, including flavonoids [11,16,38,51–60]. Among chalcones tested in combination with TRAIL, flavokawain B, isoliquiritigenin and butein have induced TRAIL-R2 expression so far [47–50]. An increase of TRAIL-R2 expression and abrogation of TRAIL-resistance in cancer cells has been attributed to various flavonoids belonging to the flavonols, flavanones, isoflavones, flavones and flavonolignans subclass. Quercetin, kaempferol (flavonols) [52–55], naringenin, 6-hydroxy-flavanone, 6-propionoxyflavanone (flavanones) [51,56], biochanin A (isoflavones) [11], apigenin, luteolin, wogonin, baicalein (flavones) [38,56–59], and silibinin (flavonolignans) [60] enhance TRAIL-mediated death in cancer cells also by increase of TRAIL-R2 mRNA and/or TRAIL-R2 protein levels.

## 3. Experimental Section

### 3.1. Reagents

Soluble recombinant human TRAIL (rhsTRAIL) was purchased from PeproTech Inc. (Rocky Hill, NJ, USA). Human recombinant TRAIL-R2/Fc chimera protein was obtained from R & D Systems (Minneapolis, MN, USA). The four chalcones: chalcone (*trans*-benzylideneacetophenone) (**CH**), isobavachalcone (2′,4′,4-trihydroxy-3′-[3′-methylbut-3′-ethyl]chalcone) (**IC**), licochalcone-A (E-3-[5-(1,1-diethyl-2-propenyl) -4-hydroxy-2-methoxyphenyl]-1-(4-hydroxypenyl)-2-propen-1-one) (**LC**) and xanthohumol (2′,4,4′-tri-hydroxy-3′-prenyl-6′-methoxychalcone) (**XH**) were obtained from Alexis Biochemicals (Lausanne, Switzerland). The compounds were dissolved in DMSO (50 mM) to a final concentration of 0.1% (*v/v*) in the culture media.

### 3.2. Cancer Cell Culture

The experiments were performed on a HeLa human cervical cancer cell line obtained from DSMZ (Deutsche Sammlung von Mikroorganismen und Zellkulturen) GmbH-German Collection of Microorganism and Cell Cultures (Braunschweig, Germany). The HeLa cells were grown in monolayer cultures in RPMI 1640 containing 10% fetal bovine serum with 4 mM l-glutamine, 100 U/mL penicillin and 100 μg/mL streptomycin. The cells were maintained at 37 °C in atmosphere with 5% CO_2_[13,39]. All reagents for cell culture were purchased from PAA Laboratories (Pasching, Austria).

### 3.3. Detection of Cell Death Using MTT Cytotoxicity Assay

The cytotoxicity was determined by the 3-(4,5-dimethylthiazol-2-yl)-2,5-diphenyltetrazolium bromide (MTT) assay as described previously [61,62]. The HeLa cells (2.5 × 10^5^/mL) were seeded 24 h before the experiments in a 96-well plate and incubated with TRAIL (100 ng/mL) and/or chalcones (25 μM and 50 μM) for 24 h. Next, the medium was removed and 20 μL MTT solutions prepared at 5 mg/mL (Sigma Chemical Company, St. Louis, MO, USA) were added to each well for 4 h. The resulting crystals were dissolved in DMSO. Controls included native cells and medium alone. The spectrophotometric absorbance of each well was measured using a microplate reader (ELx 800, Bio-Tek Instruments Inc., Winooski, VT, USA) at 550 nm. The cytotoxicity was calculated by the formula: percent cytotoxicity (cell death) = [1 − (absorbance of experimental wells/absorbance of control wells)] × 100%.

### 3.4. Lactate Dehydrogenase Release Assay

Lactate dehydrogenase (LDH) is a stable cytosolic enzyme released upon membrane damage in necrotic cells. Measurement of LDH activity was performed using a commercial cytotoxicity assay kit (Roche Diagnostics GmbH, Mannheim, Germany). The HeLa cells were treated with TRAIL (100 ng/mL) and/or chalcones (25 μM and 50 μM) for the indicated period of time. The sample solution (supernatant) was removed and LDH released from cells was detected in culture supernatants with a coupled enzymatic assay, resulting in conversion of a tetrazolium salt into red formazan product. The maximal release was obtained after treating control cells with 1% Triton X-100 for 10 min at room temperature [15,63]. The necrotic percentage was expressed using the formula: (sample value/maximal release) × 100%.

### 3.5. Detection of Apoptotic Cell Death by Flow Cytometry

Apoptosis was determined by flow cytometry using the Apoptest-FITC Kit with annexin V (Dako, Glostrup, Denmark). HeLa cells (2.5 × 10^5^/mL) were seeded in 24-well plates for 24 h prior to experimentation and then exposed to TRAIL (100 ng/mL) and/or chalcones (25 μM and 50 μM) for 24 h. After the incubation, the cells were washed twice with phosphate-buffered saline solution (PBS) and resuspended in 500 μL of binding buffer. The cell suspension (290 μL) was then incubated with 5 μL of annexin V-FITC and 5 μL of propidium iodide for 10 min at room temperature in the dark. The population of annexin V-positive cells was evaluated by flow cytometry (LSR II, Becton Dickinson Immunocytometry Systems, San Jose, CA, USA) [39,63].

### 3.6. Detection of Apoptotic Cell Death by Fluorescence Microscopy

Apoptotic cells were quantified by the fluorescence microscopy method using the Apoptotic & Necrotic & Healthy Cells Quantification Kit from Biotium, Inc. (Hayward, CA, USA) according to the manufacturer’s instruction [13,25]. The HeLa cells (2.5 × 10^5^/mL) were seeded 24 h before the experiments in a 24-well plate. TRAIL (100 ng/mL) and/or chalcones (25 μM and 50 μM) were added to cancer cells, and 24 h later the cells were washed with PBS and detached from cell culture wells by trypsin. Next, the cells were centrifuged to discard supernatant, washed with PBS and resuspended in binding buffer (100 μL/sample). To each tube there was added: 5 μL Annexin V-FITC, 5 μL Ethidium Homodimer III and 5 μL Hoechst 33342 solutions. The samples were incubated at room temperature for 15 min in the dark. After staining, the cancer cells were washed with Binding Buffer, placed on a glass slide and covered with a glass coverslip. The stained cells were observed under a fluorescence inverted microscope IX51 (Olympus, Tokyo, Japan) using filter set for FITC, TRITC and DAPI. The healthy cells (stained with Hoechst 33342) emitted blue fluorescence, apoptotic cells (stained with Annexin V-FITC and Hoechst 33342) emitted green and blue fluorescence whereas necrotic cells (stained with Ethidium Homodimer III and Hoechst 33342) emitted red and blue fluorescence.

### 3.7. Analysis of Death Receptor Expression on the Cancer Cell Surface by Flow Cytometry

The cell surface expression of death receptors TRAIL-R1 and TRAIL-R2 was detected by flow cytometry (LSR II, Becton Dickinson Immunocytometry Systems). HeLa cells (2.5 × 10^5^/mL) were seeded in 24-well plates for 24 h and exposed to chalcones (25 μM) for 24 h. Cells were then harvested using solution of trypsin and ethylenediaminetetraacetic acid, washed twice in PBS and resuspended in PBS containing 0.5% bovine serum albumin. Cells were incubated with 10 μL phycoerythrin-conjugated anti-TRAIL-R1 or anti-TRAIL-R2 monoclonal antibody (R & D Systems) at 4 °C for 45 min. After staining, the cells were washed with PBS and analyzed using flow cytometry [51,64]. The control sample (isotype control) consisted of cells in a separate tube treated with phycoerythrin-labelled mouse IgG_1_ or mouse IgG_2B_ (R & D Systems).

### 3.8. Statistical Analysis

The results are expressed as means ± SD obtained from three separate experiments performed in quadruplicate (*n* = 12). Statistical significance was evaluated using Student’s *t*-test. *p*-values < 0.05 were considered significant.

## 4. Conclusions

Targeting a TRAIL-induced apoptotic signaling pathway in tumor cells by polyphenols is one of the crucial issues in cancer chemoprevention. Chalcone, isobavachalcone, licochalcone-A and xanthohumol markedly augment the anticancer activity of TRAIL in HeLa cells. The chalcones sensitize TRAIL-resistant cancer cells by engaging extrinsic apoptotic pathway with increased expression of TRAIL-R2 receptor. The study suggests that the overcoming of TRAIL-resistance by chalcones may be one of the mechanisms responsible for their cancer preventive effects.
